# Yeast Chronological Lifespan Model as a Tool for Screening Aging Interventions

**DOI:** 10.3390/ijms27062633

**Published:** 2026-03-13

**Authors:** Pingkang Xu, Xinyu Zhang, Yuanxia Wang, Sajid Ur Rahman, Dejian Huang, Ziyun Wu

**Affiliations:** 1 School of Food Science and Bioengineering, Xihua University, Chengdu 610039, China; xpksdsg@163.com (P.X.); yuanxia_w@163.com (Y.W.); 2 Department of Food Science and Engineering, School of Agriculture and Biology, Shanghai Jiao Tong University, Shanghai 200240, China; zhangxinyu0729@sjtu.edu.cn (X.Z.); sajid.rahman@alumni.sjtu.edu.cn (S.U.R.); 3 National University of Singapore (Suzhou) Research Institute, 377 Linquan St., Suzhou 215000, China; dejian@nus.edu.sg

**Keywords:** yeast, chronological lifespan, nutrients, natural products, longevity genes

## Abstract

*Saccharomyces cerevisiae* is a useful model to understand the biochemistry and biology of aging. Yeast speeds up the aging study due to its short lifespan, well-established genetics, and simple measurement for lifespan. The chronological lifespan in yeast specifically emphasizes the survival rate of the population, providing data that offer more direct feedback on experimental treatments than replicative lifespan. The advancement of the yeast chronological lifespan assay has enabled researchers to efficiently screen numerous potential antiaging compounds and delve into aging theories. Through the integration of robust genetic screening and high-throughput technologies, the yeast model has facilitated the identification of various antiaging factors with potential applications in humans, shedding light on the genetic mechanisms of aging. Many natural products, similar to calorie restriction, have been shown to effectively extend the lifespan of yeast, a benefit that is also conserved in mammals. In this review, we highlight the nutrient factors, natural compounds, and genes that contribute to extending the yeast lifespan, as well as the genetic regulations underlying the aging process in yeast.

## 1. Introduction

**Figure 1:**
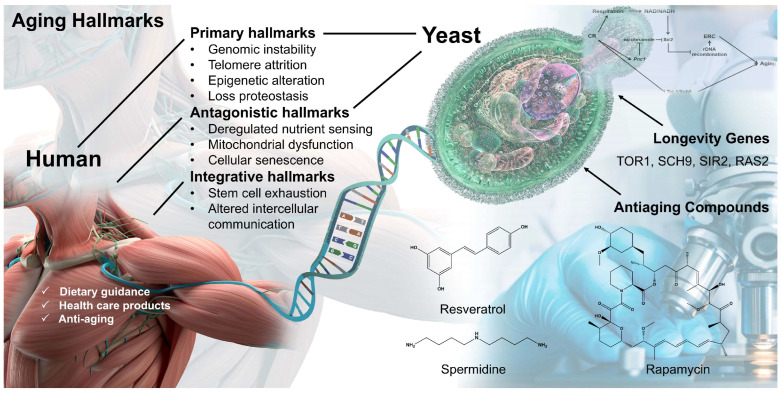
Conserved hallmarks of aging between yeast and humans.

Aging represents a gradual, insidious decline in the structural and functional integrity of multiple organ systems following reproductive maturity [1,2]. While humanity’s pursuit of antiaging interventions dates back millennia, molecular-level investigations into the genetic mechanisms of aging remain comparatively recent and incompletely elucidated. A primary challenge in aging research lies in the extensive time required to measure organismal lifespans [3]: mice and rats typically live 3–5 years, primates up to 40 years, while *Caenorhabditis elegans* and *Drosophila* survive only 2–3 weeks and 2–3 months, respectively [4]. *Saccharomyces cerevisiae* (budding yeast) offers a short chronological lifespan (CLS) (6–15 days), inherent genetic tractability, and high-throughput lifespan assays—enabling efficient mechanistic aging studies and the identification of lifespan-influencing genetic and environmental factors [5,6,7]. More importantly, many conserved hallmarks of aging between yeast and humans lead to the discovery of numerous genes and aging interventions linked to human aging from the yeast model (Figure 1).

Two primary aging models have been established in yeast research [8]. Replicative lifespan (RLS) is defined as the number of daughter cells a mother cell produces prior to senescence [9]. This model is particularly valuable for studying mitotic aging in multicellular eukaryotes, including human stem cell populations [10], as replicative aging involves asymmetric damage segregation to mother cells during budding [6]. CLS, in contrast, quantifies the ability of yeast to maintain viability during prolonged growth arrest (e.g., stationary phase) [11]. Viability is assessed by the proportion of cells capable of re-entering the cell cycle following extended quiescence [12]. The postmitotic nature of the CLS model in yeast is characterized by the cessation of cell division and entry into a non-dividing state, which is akin to postmitotic cells in higher organisms. This model is instrumental in studying cardiovascular and neurodegenerative diseases [13]. Cell cycle proteins, typically involved in cell proliferation, are repurposed in postmitotic cells, often leading to cell death upon reactivation [14]. The CLS model in yeast has also been exploited to understand the interplay between energetics and endoplasmic reticulum (ER) stress in neurodegeneration, providing insights into the postmitotic state [14].

Chronological aging in yeast serves as a relevant model for studying age-related molecular changes in the postmitotic tissues of multicellular organisms, such as neurons, skeletal myocytes and cardiomyocytes. Recent studies have expanded our understanding of the genetic, nutritional, and environmental factors that influence yeast CLS, revealing a complex interplay between these elements. The distinction between RLS and CLS is further highlighted by their distinct biological underpinnings and responses to genetic manipulations. For instance, caloric restriction, known to extend lifespan in mammals, prolongs both RLS and CLS in yeast, suggesting conserved mechanisms of lifespan regulation [15]. Additionally, the genes and pathways that influence RLS and CLS are evolutionarily conserved, underscoring the relevance of yeast as a model for understanding aging in more complex organisms [16].

Both studies focus on yeast cell survival, with CLS specifically quantifying temporal progression. Upon inoculation, yeast rapidly consumes extracellular glucose. Glucose depletion triggers a post-diauxic transition, characterized by decelerated growth and division. Mitochondrial respiration then dominates the metabolic pathway [17]. During the subsequent 2–7 days, yeast enters a quiescent stationary phase marked by low metabolic activity and enhanced stress resistance. Viability declines rapidly thereafter, culminating in death. Thus, the CLS of yeast is typically less than 30 days [18]. This process is regulated by numerous genetic pathways, including those governing lifespan through the TOR/Sch9 and Ras/adenylate cyclase/PKA signaling pathways, with a specific focus on pathways related to age-associated oxidative stress and DNA damage/repair mechanisms [19]. Yeast aging is orchestrated through a cascade of lifespan checkpoints, each governed by dedicated master regulators. Yeast chronological aging operates via network dynamics that dictate the cellular senescence pace, indicating that this process is non-programmed toward death but stems from a failure to sustain core homeostatic functions above a critical threshold. Both cell-autonomous and cell-non-autonomous mechanisms collectively govern this outcome [20]. Substantial discordance has been reported among three genome-wide screens for CLS in yeast, and gene–environment interactions—particularly differences in culture media—have been proposed as a major contributing factor [21]. CLS and RLS genetic dissection in yeast serves as a premier model for elucidating gene–environment and epistatic interactions, thereby delineating their mechanistic contributions to proteostasis and metabolic fitness [21]. In addition, the potential of natural substances in modulating yeast lifespan through various mechanisms, such as affecting autophagy, oxidant regulation, and lipid metabolism, should also not be ignored [22]. To determine the environmental factors for CLS, high-throughput screening tools have been used to uncover the conserved systems-level mechanisms such as dietary interventions [23]. Here, our focus is on the chronological aging of yeast, exploring the key aspects of CLS in yeast as a valuable tool for aging studies.

## 2. Methods for Measuring Yeast CLS

### 2.1. Traditional Colony Count Plating Method

The CLS assay in budding yeast was established by the Longo group [11,24,25]. In this protocol, day 0 marks the inoculation time point. Subsequent survival measurements are performed every 2–4 days: cultures are diluted and plated onto YPD agar and colony-forming units (CFUs) are counted after 3 days of incubation. The survival rate at day 2–3 (typically defined as 100% initial viability) serves as the reference point, with CLS quantified as the proportional decline in viability over time. CFU counts are monitored until viability drops below 1% [11].

Recently, an alternative method for assessing yeast chronological aging was proposed, combining propidium iodide (PI) staining with flow cytometry (PI-FCM) [26,27]. This approach exploits PI’s inability to penetrate viable cells due to intact plasma membranes, while dead cells exhibit membrane permeability and stain red. Coupled with flow cytometry, PI-FCM enables rapid, high-throughput, and reproducible quantification of viability—reducing labor intensity compared to CFU-based assays. However, a subsequent study revealed that PI-FCM accuracy is highly dependent on culture conditions: correlation with CFU counts is only observed in yeast aged in exhausted media, but not in water-based cultures [28].

### 2.2. High-Throughput Method Based on Outgrowth of Aging Cells

The Kaeberlein group introduced an automated viability assay for yeast CLS using the Bioscreen C MBR system, enabling longitudinal tracking of population dynamics at scheduled temporal intervals [29]. A measurement of 5 µL of aged culture is inoculated into 145 µL of YPD medium per well in a 100-well Bioscreen Honeycomb plate. The instrument incubates up to 200 samples simultaneously at 30°C with continuous agitation, recording optical density (OD) at 420–580 nm every 30 min over 24 h. Outgrowth curves are generated for each well/sample based on OD measurements, and viability is inferred from these curves. Compared with the traditional CFU plating method, this approach demonstrates reduced technical variance while being faster, less labor-intensive, and more cost-effective. Additionally, it yields supplementary physiological parameters—including doubling time, growth rate, and culture optical density—enabling a multidimensional analysis of aging yeast populations.

Kaeberlein’s method involves traditional assays that are based on plating yeast cells in a specialized stationary phase and measuring cell survival for CLS. In addition, Kaeberlein’s method measured OD values for 30 min each time. The accuracy of the time required to reach the target absorbance value is not as expected. Based on Kaeberlein’s method, we developed a more rapid and efficient high-throughput screening assay for measuring yeast CLS under various conditions [18] (Table 1). This protocol employs standard plate readers compatible with chemical or genetic libraries in 96- or 384-well formats, overcoming the limitation of Kaeberlein’s approach which requires non-standard 100-well Bioscreen Honeycomb plates. The absorbance reading duration is substantially shorter in this assay due to a higher initial concentration of yeast. An additional alteration involves raising the frequency of absorbance reading. We record the OD every 5 min, which improves the accuracy of time taken to attain the goal absorbance value, as opposed to 30 min for each OD determination in Kaeberlein’s method. The effects of environmental factors, such as the nutrients described later in the paper, can be determined by this method.

Molecular barcode sequencing (Bar-seq) and pooled strain coupled to microarray are other high-throughput methods for measuring yeast genotypes. Using Bar-seq, the effects of ~4000 gene deletions and ~12,000 pairwise genetic interactions were assessed across three nutrient-restricted environments (carbon, nitrogen, and phosphorus limitation) [30]. The methodology involved the construction of haploid prototrophic double mutant libraries using synthetic genetic array (SGA) mating and selection methods. Pooled analysis of genotypes through Bar-seq was determined to assess the role of these genes in both proliferative and quiescent cellular states. This high-throughput method assay allowed for the detection of up to 10-fold more genetic interactions in quiescent cells compared to proliferative cells, highlighting the conditional dependence of epistasis and providing a framework for expanding genome-wide genetic interaction profiling to diverse conditions and phenotypes. The importance of genetic interactions and the role of nutrient-sensing pathways, such as the target of *TOR1*, *RIM15* and *PHO85* pathways, in regulating lifespan was highlighted [30]. The pooled strain coupled to microarray approach enables high-throughput measurement of yeast CLS by assessing the viability of non-dividing yeast cells over time. This method utilizes a collection of haploid gene deletion mutants, each tagged with unique DNA barcodes, which are grown in pooled cultures and subjected to aging conditions. At various time points, viable mutants are selected, and their abundance is quantified using microarray DNA hybridization, allowing for the identification of genes that influence CLS. This technique efficiently screens a large number of mutants to uncover novel factors involved in the regulation of aging, providing insights into the genetic mechanisms that contribute to longevity [31,32].

High-throughput plating also plays a pivotal role in measuring CLS by enabling the assessment of survival across numerous strains under various conditions. This method allows for the rapid determination of survival integrals (SIs), a quantifiable parameter of CLS, using automated imaging and image analysis of agar yeast culture arrays [33,34]. They facilitate the systematic investigation of genetic and environmental factors influencing yeast aging, providing insights into the complex mechanisms of chronological aging.

At the level of protein, high-throughput methods are also applicable. A high-throughput screening approach has been used to investigate the noncatalytic functions of yeast enzymes [35]. The researchers generated knockout and catalytic mutant strains of selected genes, assessed their growth under various conditions using automated phenotypic characterization, and analyzed the data to distinguish between catalytic and noncatalytic phenotypes. This strategy revealed that a significant number of gene deletion phenotypes in yeast are not solely attributed to the loss of annotated catalytic activities, highlighting the prevalence of protein moonlighting in cellular metabolism [35].

The FY4 strain of *S. cerevisiae* has been prominently featured in the study of CLS in yeast, serving as a model organism to elucidate the complex mechanisms underlying cellular aging. Its widespread adoption stems from a combination of favorable attributes: as an isogenic derivative of the S288c reference strain, FY4 exhibits minimal genetic drift, low spontaneous mutation rates, and exceptionally reproducible growth and survival kinetics under defined culture conditions [36]. These features collectively ensure high assay fidelity, which is critical for quantitative aging studies. In the development of high-throughput protocols and programs for growth and aging phenotyping in yeast, the FY4 strain was again highlighted. The researchers adapted the growth phenotyping assay to a 384-well microplate format, validating the higher-throughput approach for phenotypic screens. The FY4 strain was used to demonstrate the reproducibility and reliability of the assay, which allowed for the accurate determination of growth parameters such as lag time, doubling time, and yield of biomass [36]. This methodological advancement has since facilitated more efficient and cost-effective studies on yeast-based systems genetics and screens, including those focused on CLS.

### 2.3. Limitations of the Yeast CLS Assay

Although CLS assay has been widely employed in aging research and has successfully identified evolutionarily conserved longevity pathways, including TOR/Sch9, Ras/AC/PKA, SNF1, sirtuins, and numerous other pro-longevity genes, its biological extrapolation as a model of mammalian aging entails significant limitations that warrant a cautious interpretation of results [1,37,38,39,40].

Standard CLS assays quantify survival as CFUs following glucose exhaustion; however, cells do not immediately enter a state of deep nutrient deprivation. Instead, they maintain elevated metabolic activity for 4–6 days by respiring endogenous reserves such as glycogen and trehalose, as well as extracellular ethanol, only gradually transitioning into a low-metabolism “quiescent” state prior to death [23]. This indicates that the early phase of CLS reflects a metabolically reprogrammed adaptive state rather than a simple model of caloric restriction.

Consequently, CLS fundamentally measures cellular tolerance to energy stress, not the progressive accumulation of molecular damage, functional decline, and systemic dysregulation that define aging in multicellular organisms. In stark contrast to human postmitotic cells, such as neurons and cardiomyocytes, which undergo rapid functional collapse within minutes under hypoglycemic conditions, yeast can survive for weeks by relying on internal energy stores, underscoring a profound divergence in bioenergetic dynamics [41]. Moreover, although yeast CLS is frequently analogized to quiescent mammalian cells, this comparison is physiologically limited due to the absence of neuronal signaling pathways in yeast [42].

CLS endpoints are often confounded by acetate accumulation in the culture medium, which triggers DNA fragmentation and other apoptosis-like features; however, the relevance of acetate-mediated cell death to physiological aging in mammals remains unestablished [43]. Notably, acetate has long been simplistically viewed as a toxic byproduct, but recent evidence suggests that it functions primarily as an alternative carbon source: elevated acetate levels induce glycogen and trehalose accumulation, promote mitochondrial fragmentation, and exert pro-aging effects independent of extracellular pH; furthermore, buffering the medium fails to extend CLS, thereby refuting the notion that acidosis alone drives mortality [23,44]. Moreover, the CLS assay is based on the implicit assumption that all cells enter a quiescent state upon glucose exhaustion, but the experimental evidence shows that a subset of cells can sustain limited proliferation by scavenging nutrients released from dead cells, a phenomenon known as necrotrophy or “cannibalism”, thereby confounding the accurate assessment of true quiescent survival [45]. Critically, CLS relies on the ability of cells to form colonies upon re-plating onto nutrient-rich media; thus, cells that have lost their proliferative capacity but remain metabolically viable (e.g., G_0_-arrested or senescence-like cells) are misclassified as dead, potentially leading to systematic underestimation of actual viability [23,41]. Collectively, these findings indicate that CLS represents a complex integration of carbon source switching, metabolic adaptation, oxidative stress, and regulated cell death, not merely starvation tolerance.

Yeast lacks the insulin/IGF-1 signaling pathway, which in mammals profoundly influences lifespan through the regulation of mTOR and AMPK, and reduced circulating IGF-1 levels are consistently associated with exceptional human longevity [46,47]. Although the mTOR pathway is evolutionarily conserved, its functional outputs diverge across species: rapamycin-mediated TOR inhibition suppresses mitochondrial respiration in mammals but enhances it in yeast [48]. Furthermore, mammals possess a single TOR gene that contributes to both TORC1 and TORC2 complexes; *S. cerevisiae* harbors two paralogs (*TOR1* and *TOR2*) with distinct complex specificities [49]. As a screening platform, CLS is therefore constrained by an artificial energetic boundary defined by the initial glucose availability, limiting its capacity to model antiaging interventions requiring prolonged action or multi-target synergy.

As a unicellular organism, yeast entirely lacks multicellularity, cellular differentiation hierarchies, and sophisticated intercellular communication networks. In contrast, a substantial fraction of the human protein-coding genes govern neural, immune, and stem cell functions that are exquisitely dependent on tissue microenvironments. Mammalian aging is inherently non-cell-autonomous: senescent cells drive inflammaging, immune surveillance decline, and bystander effects via the senescence-associated secretory phenotype (SASP), and can also transfer functional proteins directly to neighboring cells—such as NK cells and neurons—through cytoplasmic bridges (e.g., tunneling nanotubes) in a process termed intercellular protein transfer (IPT), thereby modulating microenvironmental homeostasis and immune clearance [50]. These systemic aging hallmarks, driven by stem cell exhaustion, chronic inflammation, and intercellular signaling imbalances, cannot be recapitulated in the isolated yeast CLS system and are unlikely to model the deleterious paracrine effects observed in mammalian tissues [4].

In light of these considerations, while CLS remains valuable for mechanistic discovery, its translational relevance to human aging should be approached with caution and validated in multicellular models that incorporate tissue architecture, systemic physiology, and cellular heterogeneity. CLS is best positioned as an initial screening tool rather than a comprehensive model of organismal aging. Future studies must interpret its biological significance within an integrative, cross-species framework.

## 3. Nutrients and Yeast Longevity

### 3.1. Carbohydrates

Glucose restriction, commonly termed calorie restriction (CR) or dietary restriction (DR) in yeast aging models, significantly extends yeast CLS. The standard CR protocol reduces the glucose concentration from 2% to 0.5% in the medium. However, studies report that further restriction to 0.05% glucose (extreme CR) enhances longevity beyond standard CR, as does the water-transfer method (transferring cells from 2% glucose to water). Although extreme CR causes malnutrition in higher organisms [51], it remains a validated longevity intervention in yeast. Current research indicates that glucose-mediated longevity involves the TOR and Ras/cAMP/PKA nutrient-signaling pathways. The FY4 strain was instrumental in identifying major quantitative trait loci (QTL) associated with aging, which were found to be dependent on the type and concentration of the carbon sources available for growth. The *RIM15* gene was recognized as a key regulator of aging in low glucose environments, providing additional evidence for the critical role of nutrient-sensing pathways in the regulation of longevity under caloric restriction [34].

Glycerol, a non-fermentable carbon source that bypasses glycolysis, extends the CLS [52,53,54]. Recent evidence indicates that acetate accumulation—rather than direct toxicity—drives chronological aging under standard culture conditions Conversely, glycerol did not impair CR-induced CLS extension, suggesting that it modulates chronological aging via stress resistance pathways—specifically, enhancing osmotic stress tolerance and modulating cellular redox balance [54]. Recent work revealed that WT cells accumulate ethanol and rapidly deplete glycerol, whereas long-lived mutants (*tor1Δ*, *sch9Δ*, *ras2Δ*) accumulate glycerol after initial ethanol depletion. This indicates that TOR/Sch9 inhibition triggers a metabolic switch from ethanol biosynthesis/release to glycerol biosynthesis and release [55,56,57].

### 3.2. Amino Acid Supplementation

The concept that diets with identical caloric values but different compositions might influence longevity differently was first introduced in mice studies [58,59]. This idea was subsequently explored in *Drosophila melanogaster* and extended to a range of organisms, from yeast to humans [51,60,61,62]. Research has shown that both restricting and increasing protein and amino acid intake can play roles in delaying the aging process [63,64]. Amino acids are essential nutrients recycled via autophagy in yeast [65]. Naturally, yeast is prototrophic and synthesizes most amino acids from simple carbon/nitrogen sources, whereas laboratory strains (e.g., *S. cerevisiae* BY4742 MATα *his3Δ1 leu2Δ0 lys2Δ0 ura3Δ0*) are typically auxotrophic. This auxotrophy confers nutrient-dependent growth useful for genetic manipulation, commonly resulting from deletions in amino acid/nucleotide biosynthesis genes.

Suboptimal supplementation of auxotrophic complementing amino acids (EAAs) suppresses the final biomass yield, impairs oxidative stress tolerance, induces premature G2/M arrest, and accelerates chronological aging [66]. Consistent with this, a reduced total amino acid availability—encompassing EAAs—in growth media similarly shortens CLS [29]. In non-dividing auxotrophs, leucine or uracil deprivation triggers exponential viability decline with a half-life < 2 days [67]. Notably, survival outcomes in such auxotrophs depend on the carbon source during starvation—not on the growth medium carbon source [68]. Methionine restriction enhances autophagy, a cellular self-digestion process, and increases the number of cells with acidic vacuoles, which is crucial for longevity [69].

Nonessential amino acids (NEAAs) modulate yeast lifespan. We observed that methionine and glutamic acid—NEAAs for yeast—most potently extend CLS. Restricting methionine and/or elevating glutamic acid yielded longevity not merely due to the reduced acetic acid production and media acidification. Critically, low methionine, high glutamic acid, and glucose restriction independently and additively extended the lifespan, a phenotype unenhanced by medium buffering (pH 6.0) [61].

Branched-chain amino acids (BCAAs)—leucine, isoleucine, and valine, all essential—extend CLS in both autophagy-competent and autophagy-deficient yeast (e.g., *atg1Δ*, *atg7Δ*, *atg11Δ*) grown in minimal medium [65]. These BCAAs extend CLS through the general amino acid control (GAAC) pathway: a reduced BCAA concentration activates GAAC and shortens CLS, whereas elevated BCAA levels inhibit GAAC and extend CLS [65]. Proline, which is recognized not only as a fundamental building block but also as an energy source and stress shield, has been shown to increase the CLS through a mechanism that depends on proline oxidase (PUT1) [70], while serine depletion has been associated with an increased lifespan. Furthermore, restricting the intake of valine and threonine might support the longevity effect of calorie restriction by inhibiting the TOR-Sch9 pathway through the Sch9 phosphorylation [7].

### 3.3. Other Nutrients

In yeast, ammonium (NH_4_^+^) functions as the principal inorganic nitrogen source, fueling both anabolic and catabolic nitrogen metabolism through its assimilation into glutamate and glutamine—the central donors for transamination reactions [71]. Nitrogen source selection is governed by nitrogen catabolite repression (NCR), which prioritizes preferred sources like NH_4_^+^. However, recent studies reveal that extracellular NH_4_^+^ acts as a pro-aging factor: its accumulation impairs viability in stationary-phase cells, thereby shortening CLS [72]. Reducing the NH_4_^+^ concentration extends CLS in amino acid-restricted media. Conversely, increasing the (NH_4_)_2_SO_4_ concentration (0.5% → 1%), with or without amino acid restriction, reduces the viability. Furthermore, adding NH_4_^+^ to aging cells transferred to water significantly shortens CLS, confirming that NH_4_^+^ alone induces viability loss. This NH_4_^+^-mediated cell death involves conserved pathways: PKA and TOR/Sch9 [73,74].

Utilizing a quiescence profiling technology, it has been shown that auxotrophic markers, such as those distinguishing strains 4741 and 4742, media composition—including amino acids and ammonium (NH_4_^+^)—along with TOR signaling, collectively influence CLS [33]. The study highlighted that the availability of specific amino acids and the balance of NH_4_^+^ in the growth medium significantly impact yeast survival, with certain auxotrophies showing reduced CLS under nutrient-limited conditions. Furthermore, it demonstrates that TOR signaling, a key regulator of cellular growth in response to nutrient availability, plays a crucial role in modulating the effects of these genetic and nutritional factors on yeast longevity. These findings underscore the complex interplay between genetic background, nutrient environment, and cellular quiescence in determining the CLS of yeast [33].

In addition, low-quality nitrogen sources like c-aminobutyric acid (GABA) extend CLS compared to high-quality sources such as glutamine. Genetic analysis identified 473 gene mutants with altered CLS under these conditions, highlighting cellular processes like autophagy, mitochondrial function, and cell cycle control. Key transcription factors, including Msn2, Msn4, and Ste12, were found to regulate CLS extension, with Ste12 linking nutrient signaling to cell survivorship and cell cycle arrest, suggesting a conserved role in longevity across nitrogen sources [75].

## 4. Natural Products for Lifespan Extension

Perhaps the most effective antiaging intervention is CR. Recent research suggested that the beneficial health effects of CR might be attained by a compound that alters the activity of some evolutionarily conserved longevity proteins in response to nutrient availability [76]. The compound could act as a “CR mimetic” by delaying age-associated diseases and extending lifespan without requiring reduced food intake [77]. There are a number of natural products that have been reported to have a lifespan-extending capacity or antiaging benefit in mammalian animals. The antiaging capacity of most compounds was first found in simple organisms, such as yeast, *C. elegans*, and *D. melanogaster*. Though only a few compounds could have potential effects of CR in mammals, the pre-screening and mechanism exploration in simple organisms provide valuable direction in aging research. Here we list some of the antiaging substances that are effective in yeast models and whose functions extend to higher animals.

### 4.1. Rapamycin

Rapamycin (Figure 1) was originally isolated from a soil-derived strain of *Streptomyces hygroscopicus* on Easter Island and identified by Brazilian scientists as a potent antifungal agent [78]. After initial studies as an immunosuppressant (prior to the elucidation of its mechanism), rapamycin was approved by the FDA in 1999 for post-transplant therapy [79].

Rapamycin binds FKBP12, forming a complex that inhibits mTOR Complex1 (mTORC1) by direct binding. mTOR (mechanistic target of rapamycin) was historically called FRAP, RAFT, RAPT1, or SEP, but is now universally accepted following the identification of *TOR1* and *TOR2* as rapamycin-sensitive growth regulators in *S. cerevisiae* [80]. The mTOR signaling axis integrates diverse environmental inputs to coordinate fundamental cellular processes—including anabolic metabolism, protein synthesis, lipid biosynthesis, energy balance, autophagic flux, lysosomal biogenesis, and cytoskeletal dynamics [64]. Dysregulation of this pathway is causally linked to multiple age-associated pathologies, such as cancer, obesity, type 2 diabetes, neurodegenerative disorders, and organismal aging [81].

Rapamycin extends lifespan in simple model organisms and mammals. At low concentrations, rapamycin increases the yeast CLS (Table 2). Rapamycin extends the CLS of yeast through the inhibition of the TOR pathway and thus causes the induction of stress-responsive processes, such as the upregulation of autophagy [65,80]. Rapamycin remains one of the few compounds shown to extend the maximum lifespan in mammals. The National Institute on Aging’s Interventions Testing Program (ITP)—a multicenter initiative—demonstrated that rapamycin-mediated mTOR inhibition extends both the median and maximal lifespans in genetically diverse mice [82,83,84]. This longevity benefit is observed in males and females when treatment commences at 600 days of age, a stage corresponding to approximately 60 years in humans. Although direct translation to human aging remains unproven, these findings provide a compelling rationale for advancing mTOR-targeted strategies to mitigate age-related pathologies, even when initiated during midlife [84].

Early human trials reported serious side effects of rapamycin, including hyperlipidaemia, hyperglycaemia, anemia, and stomatitis [79]. As an immunosuppressant, it increases susceptibility to opportunistic infections [89]. Chronic rapamycin treatment impairs glucose homeostasis, inducing diabetes-like symptoms such as reduced glucose tolerance and insulin insensitivity [90]. However, the optimal dosage and treatment duration remain undefined; low bioavailability and atypical pharmacokinetics likely limit tissue drug exposure, thereby attenuating adverse effects.

To mitigate these side effects, alternative treatment regimens have been explored. Research indicates that intermittent administration of rapamycin can extend lifespan and health span in mice without the negative impacts on glucose homeostasis and the immune system observed with daily administration. Furthermore, co-treatment with metformin, a drug used to treat type 2 diabetes, has been shown to normalize insulin sensitivity and reduce complications of metabolic syndrome in type 2 diabetic mice treated with rapamycin. This co-treatment strategy could potentially counteract the hyperglycemic effects of rapamycin, providing a clinical approach to manage the diabetes-like symptoms induced by chronic rapamycin treatment [91].

Ethical considerations are also paramount in the development and use of such interventions. The potential for rapamycin to extend health span and delay age-related diseases is weighed against the risks of side effects, including the impact on glucose homeostasis and immune function [92]. It is crucial to ensure that the benefits of such treatments are accessible to all, regardless of socioeconomic status, and that the autonomy of individuals in choosing to undergo treatment is respected. Additionally, the long-term effects and potential for overuse or misuse of these interventions must be carefully considered to prevent unintended consequences on public health.

### 4.2. Resveratrol

Resveratrol (Figure 1) and its structural analogs are polyphenolic phytoalexins naturally produced by diverse plant species, including Japanese knotweed (*Fallopia japonica*), grapes, berries, and peanuts. Preclinical evidence across cellular and animal models indicates that resveratrol exerts protective effects in pathophysiological contexts relevant to human aging and age-associated disorders, notably cancer, cardiovascular disease, chronic inflammation, type 2 diabetes, and ischemic injury [93,94,95,96]. It extends RLS in *S. cerevisiae* and prolongs lifespan in diverse organisms (Table 3). However, RLS extension (up to 100% at >10 µM) occurs without CLS extension even at 100 µM in the short-lived PSY316AT strain [78]. Although numerous studies report the longevity-enhancing effects of resveratrol, robust evidence for lifespan extension in mammals remains lacking. Resveratrol remains a candidate antiaging compound due to its proposed activation of sirtuins (NAD-dependent histone deacetylases, HDACs). In mammals, seven SIRT isoforms (SIRT1–SIRT7) exhibit distinct biochemical activities and subcellular localizations: nucleus (SIRT1, SIRT2, SIRT6), nucleolus (SIRT7), cytoplasm (SIRT1, SIRT2), and mitochondria (SIRT3–SIRT5). SIRT1 regulates key processes including metabolism, cell survival, DNA repair, and apoptosis [97,98].

Both natural (e.g., resveratrol, butein, fisetin, quercetin) and synthetic (SRT1460, SRT1720, SRT2183) sirtuin-activating compounds (STACs) enhance SIRT1 catalytic efficiency in vitro by reducing the Michaelis constant for acetylated substrates and nicotinamide adenine dinucleotide (oxidized form) (NAD^+^), thereby promoting cell survival through SIRT1-mediated deacetylation of p53 [99]. In yeast, resveratrol extends lifespan by mimicking caloric restriction via the activation of Sir2—the functional ortholog of mammalian SIRT1—thereby enhancing genomic stability. Although the physiological relevance of direct SIRT1 activation by STACs has been contested [108,109], recent biochemical reconstitution assays from the Sinclair group confirm that resveratrol and related STACs can allosterically stimulate SIRT1 activity in vitro, but strictly in the context of specific peptide substrates containing a bulky hydrophobic residue at the +1 position relative to the acetyl-lysine [109].

Ever since the functions of resveratrol were reported, there has been no end to the discussion about the actual functions of this substance. Although in vitro and animal studies provide strong evidence that resveratrol can activate SIRT1, there are serious doubts about the applicability and durability of this action in human physiology. The physiological significance of these discoveries is called into question because, for instance, the levels of resveratrol needed to activate SIRT1 in vitro are far higher than those that can be obtained in vivo. Furthermore, AMPK activation and mTOR inhibition are two additional cellular pathways that may contribute to the advantages of resveratrol, suggesting that its effects are not exclusively mediated by SIRT1. Furthermore, even while resveratrol seems to benefit model organisms, human health may not necessarily benefit from these findings.

While the direct activation of SIRT1 by resveratrol remains a plausible hypothesis based on in vitro and early animal research, the clinical relevance of this effect in humans is uncertain. The mixed results from animal and human studies, coupled with the existence of alternative pathways through which resveratrol may exert its effects, suggest that the relationship between resveratrol and SIRT1 activation is more complex than initially proposed. Further research, particularly with more advanced clinical trial designs and better bioavailability strategies, is needed to clarify the role of SIRT1 in mediating the health benefits of resveratrol.

### 4.3. Spermidine

Spermidine, a natural polyamine, has been extensively studied for its role in modulating CLS in yeast, and its effects appear to be mediated through the regulation of autophagy, inflammation, and cellular metabolism. Protection of nuclear DNA: At the molecular level, spermidine has been shown to induce autophagy by upregulating the expression of autophagy-related genes (Atg) such as *Atg7*, *Atg15*, and *Atg11* specifically through mitophagy. This induction of autophagy is critical for spermidine’s antiaging effects, as knockout of these genes abolishes the lifespan extension induced by spermidine [110]. Spermidine can reduce the spontaneous *CAN1* locus mutation rate, protecting yeast nuclear DNA under stresses such as oxidative stress [111,112]. Additionally, spermidine initiates autophagy by inhibiting protein acetylation, reducing the expression of EP300, an acetyltransferase that promotes the acetylation of autophagy-essential proteins [113]. By decreasing the availability of acetyl-CoA, spermidine also enhances deacetylation, which is crucial for the regulation of gene expression and cellular metabolism [114].

The conservation of spermidine’s effects across different species is particularly noteworthy. Studies have shown that spermidine can extend the lifespan of yeast, nematodes, flies, and mice in an autophagy-dependent manner [114]. This conservation suggests that the pathways targeted by spermidine are evolutionarily conserved, offering promise for the development of therapeutic interventions against aging and age-related diseases in humans. Epidemiological evidence also supports the potential health benefits of spermidine, as an increased dietary intake of spermidine has been associated with reduced cardiovascular and cancer-related mortality in humans, further highlighting its potential as a dietary intervention for promoting health and longevity [115,116].

### 4.4. Other Natural Compounds

Flavonoids are a group of natural compounds with different phenolic structures and are mainly available in vegetables, fruits, grains, tea and wine. In this regard, quercetin, a flavonoid found in numerous plants, exhibited an increase in lifespan when tested on the lifespan of *S. cerevisiae* [117]. However, when cells that were highly susceptible to oxidative and apoptotic stress were treated with quercetin, it led to a significant reduction in ROS levels, enhanced stress resistance, and an increase in mitochondrial membrane potential [118]. Another flavonoid, known as 4,4′-dimethoxychalcone, derived from the Japanese ashitaba plant, has shown the ability to extend CLS in *C. cerevisiae* with a dose of 50 µM [119]. The authors showed that 4,4′-dimethoxychalcone extends CLS by reducing ROS levels in WT yeast cells and triggering autophagy via Gln3 inhibition. Particularly, 4,4′-dimethoxychalcone greatly influenced amino acid metabolism through a mechanism independent of the Rapa/mTOR pathway [119].

Annurca apple extracts, which mainly comprise polyphenolic compounds, especially phloridzin (a flavonoid glucoside making up about 20%) [120], and cocoa extracts rich in polyphenols have recently been examined and shown to effectively prolong the yeast CLS [121]. The Annurca apple extracts promoted yeast CLS, exhibiting antioxidant activity mainly through the Sir2 and Sod1/Sod2 activities [120]. On the other hand, the cocoa extract increased longevity, but did so in an Sod2-independent manner [122]. Astaxanthin, a keto-carotenoid mostly found in orange- and red-pigmented plants, significantly prolonged lifespan by neutralizing ROS, reducing lipid peroxidation, and alleviating apoptotic factors. This was observed specifically in mutants that were either anti-apoptotic-deficient or antioxidant-deficient [123].

While certain phenolic compounds did not extend yeast lifespan, they showed potential in improving other aging-associated phenotypes. For instance, chebulinic acid, a type of polyphenol, and boeravinone B, a rotenoid flavonoid, restored cell viability in the presence of stress caused by malachite green. Both phenols effectively reduced the H_2_O_2_ levels triggered by this malachite green, and also declined the activities of enzymes such as catalase, superoxide dismutase (SOD), and apoptotic caspase [124]. Flavonoids extracted from citrus peels, like tangeretin and nobiletin, along with their 5-demethylated derivatives, 5-demethylnobiletin (5-DN) and 5-demethyltangeretin (5-DT), reduced ROS levels and lipid peroxidation. Additionally, they boosted stress resistance to different stress agents such as H_2_O_2_, CCl_4_, and CdSO_4_, largely due to the activity of catalase [125]. Moreover, *Rhodiola rosea* extract extended the yeast lifespan, but it decreased the yeast’s resistance to oxidative stress and did not stimulate any of the primary antioxidant enzymes. Taken together, these results reveal the potential role of other, yet-to-be-identified signaling pathways in affecting compound longevity [126].

**Figure 2:**
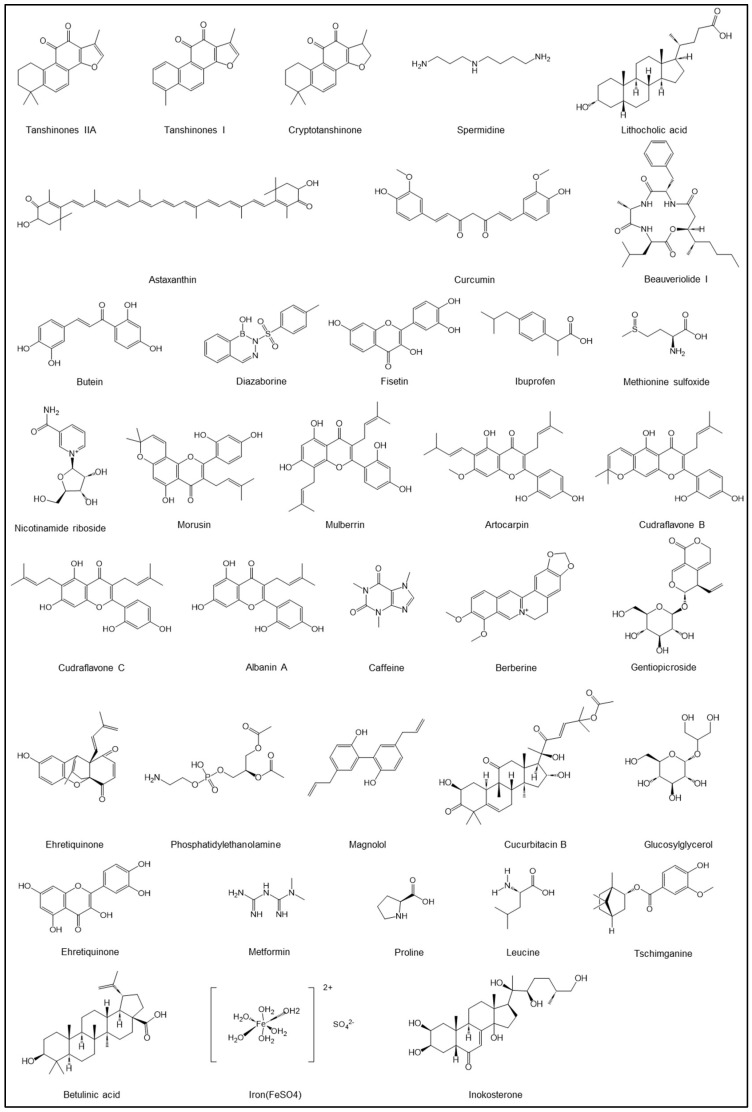
Chemical structure of natural compounds with yeast lifespan extension activity.

Many of these natural compounds were traditionally used as medicine or were previously known for their potential beneficial effects on health and yeast lifespan (Table 4 and Figure 2). Therefore, it is encouraging to observe that these natural compounds can indeed increase the lifespan or improve the metabolism of *S. cerevisiae*.

## 5. Longevity Genes

Alterations in nutrient composition and nutrient-sensing pathway activity extend lifespan by protecting against age-related diseases and reducing the incidence of age-related functional decline across species, from yeast to humans. Evolutionarily conserved nutrient-sensing signaling pathways are therefore prioritized as key targets for antiaging interventions [1]. This section focuses on central nutrient-sensing pathways in yeast CLS and their roles in lifespan regulation.

### 5.1. TOR/Sch9 Pathway

TOR kinase and its downstream effector Sch9—a functional ortholog of mammalian ribosomal S6 kinase —are evolutionarily conserved from yeast to humans, where they integrate nutrient and growth factor signals to regulate cell growth, metabolism, stress resilience, and aging [1]. While budding yeast harbors two TOR paralogs (*TOR1* and *TOR2*), metazoans possess a single TOR gene [156]. Across eukaryotes, TOR functions within two structurally and functionally distinct complexes. In *S. cerevisiae*, TORC1 (rapamycin-sensitive) contains either *TOR1* or *TOR2* in complex with Kog1 (ortholog of mammalian Raptor), Lst8, and Tco89, and governs ribosome biogenesis, translation initiation, and CLS through conserved effectors [157,158,159]. TORC2 (rapamycin-insensitive) exclusively incorporates *TOR2* together with Lst8, Avo1 (Rictor ortholog), Avo2, Avo3 (Sin1 ortholog), and Bit61, and regulates actin cytoskeleton polarization, cell wall integrity, and sphingolipid homeostasis [157,158,159]. In budding yeast, *TOR1* is non-essential for viability as *TOR2* substitutes for *TOR1* in TORC1 under *TOR1* deficiency. Conversely, *TOR2* is essential for viability since it serves as an indispensable TORC2 component, and *TOR1* cannot replace *TOR2* in TORC2 [160,161,162,163]. Overall, both TOR complexes and their rapamycin sensitivities are conserved from yeast to humans. Sch9, an TORC1 substrate, functions analogously to mammalian TORC1 substrate S6 kinase 1 (S6K1), not mTORC2 substrate PKB/Akt. *sch9Δ* mutants exhibit a small-cell phenotype (60% of wild-type volume) and slow growth, yet display strong stress resistance and extended lifespan [164]. Genetic ablation or pharmacological inhibition of *SCH9* or *TOR1* robustly extend lifespan in both chronological and replicative aging paradigms in yeast. However, sch9Δ causes more robust CLS extension than *tor1Δ*, and *tor1Δ sch9Δ* and *sch9Δ* show no significant CLS differences [54,165]. These findings indicate that *TOR1* deficiency further reduces Sch9 activity, supporting Sch9 as the primary yeast TORC1 substrate [54,166].

Attenuation of *TOR1* or Sch9 activity extends CLS downstream of dietary restriction. During chronological aging, the TORC1–Sch9 and Ras–cAMP–PKA signaling axes converge on the inhibition of Rim15, a key protein kinase that governs nuclear translocation of the stress-responsive transcription factors Msn2/4 and Gis1. Suppression of TORC1–Sch9 signaling further reduces mitochondrial reactive oxygen species (ROS) generation and potentiates cytoprotective responses, collectively driving longevity [1]. TOR also regulates general transcriptional activity. Particularly, one of its targets, Maf1, helps to inhibit the activity of DNA Pol III under nutrient deprivation conditions. Interestingly, the increased lifespan observed in yeast, worms, and flies can be attributed to Maf1, emphasizing its critical role in transcriptional regulation. This implication becomes clear when considering that the removal of Maf1 results in restraining DNA Pol III levels, leading to a decreased lifespan [167]. The effect of disrupted transcription becomes obvious through the investigation of single-cell gene expression patterns, which uncover an amplified variability in intercellular gene expression as the aging process unfolds [168]. In addition, alterations affecting subunits of the SAGA complex (Spt-Ada-Gcn5 acetyltransferase), including *DUB* and *SPT7*, further confirm its significance. This multiprotein complex, conserved across yeast and humans, arranges the acetylation and deubiquitination of both histone and non-histone proteins. Interestingly, these mutations contribute to the extension of both the CLS and RLS of yeast [169].

### 5.2. Ras/AC/PKA Pathway

In glucose-rich conditions, the budding yeast Ras/AC/PKA pathway is activated. Key components include guanosine triphosphate/guanosine diphosphate (GTP/GDP)-binding proteins (Ras1 and Ras2), a GTP-GDP exchange factor (Cdc25), GTP hydrolysis factors (Ira1 and Ira2), adenylate cyclase (AC, Cdc35/Cyr1), phosphodiesterases (Pde1 and Pde2) that catalyze cAMP hydrolysis, a PKA regulatory subunit (Bcy1), and PKA catalytic subunits (Tpk1, Tpk2, Tpk3) [37,38].

Yeast *RAS1* and *RAS2* are functional orthologs of mammalian *RAS* proto-oncogenes [170]. Both Ras1 and Ras2 act upstream of Cyr1 (AC) and exhibit partial functional redundancy in regulating cell growth, pseudohyphal differentiation, stress resilience, and CLS [11]. Ras proteins undergo a nucleotide-dependent conformational switch, cycling between an inactive GDP-bound state (Ras-GDP) and an active GTP-bound conformation (Ras-GTP). Under glucose-replete conditions, Ras activation promotes the association of Ras-GTP with adenylate cyclase (Cyr1), triggering cAMP synthesis. This cycle is positively regulated by guanine nucleotide exchange factors—Cdc25 and Sdc25—which catalyze GDP release and GTP loading, and negatively controlled by GTPase-activating proteins Ira1 and Ira2, which accelerate intrinsic GTP hydrolysis to restore the inactive state. *RAS1* deletion slightly reduces CLS, while *RAS2* deletion doubles CLS [11,170]. *RAS2* deletion enhances heat and oxidative stress resistance [11].

Ras proteins directly stimulate AC activity, and loss-of-function mutations in *CYR1*—the gene encoding yeast AC—extend CLS [171]. *CYR1* catalyzes the conversion of ATP to cAMP, serving as a central node in glucose-sensing and stress-responsive signaling. Notably, genetic ablation of the mammalian ortholog Adcy5 (encoding AC5) extends median lifespan by approximately 30% in mice and confers protection against oxidative damage, apoptosis, and age-related bone loss [172].

In *S. cerevisiae*, the cAMP-PKA holoenzyme consists of a single regulatory subunit, Bcy1, and one of three functionally distinct catalytic isoforms encoded by TPK1, TPK2, or TPK3 [173,174]. Intracellular cAMP levels are regulated through two routes: Ras1/Ras2 GTP-bound proteins (active) directly interact with Cyr1 to stimulate cAMP production, and the Gα protein Gpa2. Elevated cAMP binds to the PKA regulatory subunit (Bcy1), causing PKA (inactive) to dissociate from catalytic subunits (Tpk1, Tpk2, Tpk3). The liberated catalytic subunits (active) then phosphorylate target proteins to influence the cell physiology [175]. During chronological aging, dietary restriction reduces Ras/AC/PKA signaling, thereby activating the stress-response transcription factors Msn2/Msn4 and Gis1. This leads to the upregulation of stress-resistance genes, including *SOD2* [11]. Moreover, *TDH2* gene deletion, responsible for encoding glyceraldehyde 3-phosphodehydrogenase, is an important enzyme in glucose metabolism for both gluconeogenesis and glycolysis, and can stabilize the increased sensitivity to DNA damage resulting from HDAC dysfunction. This deletion also leads to decreased intrachromosomal recombination and has an effect on the RLS. These results suggest that Ras signaling serves as an intersection point between glucose metabolism, DNA stability, and longevity [176].

### 5.3. Sirtuins

Sirtuins constitute an evolutionarily conserved family of NAD^+^-dependent deacylases that modulate aging across eukaryotes. In *S. cerevisiae*, Sir2—a founding member of this family—functions within the silent information regulator (SIR) complex, which includes *Sir1*, *Sir3*, and *Sir4*, to establish transcriptional silencing at the mating-type loci, telomeres, and ribosomal DNA. Notably, overexpression of *Sir-2.1*, the *C. elegans* ortholog of yeast *SIR2*, extends lifespan in nematodes, an effect recapitulated by its *D. melanogaster* counterpart, *dSir2* [39,40].

Research has demonstrated that overexpressing Sir2 can increase the CLS of yeast [104]. For instance, overexpressing Sir2 has been demonstrated to increase the survival of yeast cells in the stationary phase by improving metabolic efficiency and oxidative stress resistance, which in turn increases their CLS by enhancing their ability to withstand nutrient depletion and other stresses. Additionally, knocking out the Sir2 gene, or sir2 deletion, results in a significant decrease in CLS, especially in situations of oxidative stress or nutrient deprivation, suggesting that Sir2 is essential for prolonging CLS [104,177].

In addition to Sir2, yeast has other sirtuin homologs, such as Hst1, Hst2, Hst3, and Hst4, which are also involved in regulating lifespan. These homologs can compensate for Sir2 in some functions, and their activity can also influence CLS. Hst1 has been shown to regulate histone deacetylation and contribute to chromatin silencing, similar to Sir2, and it may also impact the yeast CLS. Hst3 and Hst4 play roles in DNA repair and stress responses, which may impact CLS indirectly by maintaining genomic stability. While Sir2 has been the most studied in relation to CLS, these homologs also contribute to the overall aging process in yeast, and their interactions and redundancies with Sir2 may help fine-tune the regulation of CLS [7,178].

### 5.4. Other Genes Involved in Yeast CLS

While many model organisms, such as budding yeasts, nematodes, flies, and rodents, contribute to aging research, many CLS studies using yeast have also been conducted. In addition to the TOR/Sch9 pathway, the Ras/AC/PKA pathway, and the sirtuins family, many other genes are reportedly involved in CLS extension in yeast. The details of each gene and their mechanisms of action are shown in Table 5.

## 6. Conclusions

In conclusion, yeast chronological aging has proven to be a valuable research tool for investigating the intricate process of aging. Based on primary, antagonistic and integrative hallmarks, the homology of genes between yeast and humans, or the similarity of intracellular biochemical pathways, opens a window for drawing general conclusions about aging mechanisms. Researchers can easily manipulate the genetic and environmental factors of yeast, making it an ideal model organism. The use of various methods to measure the CLS of yeast, such as traditional colony count plating, flow cytometry, and high-throughput methods, has led to a better understanding of the aging process. The accessible gene manipulation techniques allow researchers to better understand the molecular mechanisms underlying aging and identify potential interventions. These methods have facilitated faster, more efficient exploration of aging in yeast and increased the potential for future discoveries. Furthermore, as a screening platform for antiaging interventions, yeast studies have significant potential for identifying compounds and pathways that could be translated into clinical research.

Yeast studies have demonstrated the effects of various nutrients on lifespan, such as the importance of amino acids and carbohydrates. Through calorie restriction or amino acid composition regulation, these nutrients play a crucial role in longevity. Their levels could be adjusted through dietary changes, which could potentially impact aging in higher organisms. Through yeast studies, researchers have also explored the effects of natural products such as resveratrol and rapamycin, which could influence aging through different mechanisms, including regulating the life cycle of cells and activating antioxidant activity. This research could aid in discovering new interventions to improve human health and prolong lifespan. Additionally, yeast studies have identified key evolutionary conserved signaling pathways involved in aging, such as the TOR/Sch9 pathway, Ras/AC/PKA pathway, and Sirtuins. These pathways are conserved in higher organisms, suggesting their relevance to human aging as well. Identifying these key molecular mechanisms and their role in aging could help researchers develop new antiaging therapies and prevent age-related diseases in humans that target these pathways. While yeast models have provided valuable insights into aging, it is also important to realize that there are limitations to their applicability to human aging research. The effects of genes on a unicellular organism’s cell and a cell forming a larger body may differ due to complications from multicellularity and environmental conditions. Human longevity does not measurably depend on the reproductive capacity of somatic cells, unlike yeast, where reproductive capacity is a key factor in lifespan determination. Cellular senescence in multicellular organisms involves the acquisition of senescence-associated secretory phenotype traits, which are not considered in yeast models. Additionally, yeast cells have constantly active telomerase, which does not protect them against the loss of reproductive potential, unlike human cells. In brief, by dialectically and reasonably using yeast as a model organism, life-related factors can be quickly screened out. In light of these findings, the yeast model offers a promising avenue for continuing aging research, with the potential to uncover new information about how biological processes contribute to our understanding of aging and longevity.

## Figures and Tables

**Table 1:** Assessing and contrasting two strategies for determining yeast CLS: traditional CFU plating and modern high-throughput methodology.

	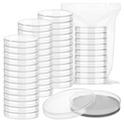 CFU Plating Method (96 Samples)	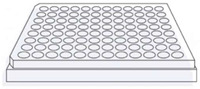 High-Throughput Method (96 Samples)
Culture dishes used	>96 culture dishes	One 96-well plate
Medium amount	>2000 mL YPD medium	10 mL YPD medium
Time consumption for medium and sample plating	>5 h	0.5 h
Incubation time	>48 h	<24 h
Recording way	Manual count	Mechanical reader
Quantitative data	CFU	Lag time, growth rate, doubling time, survival, OD
Termination of detection	Until all cells died	Until the OD value does not reach predefined number within a scheduled time (usually less than 24 h)

**Table 2:** The effect of rapamycin on yeast lifespan.

Strain	Medium	Dose/Treatment Time	Lifespan Effect	Reference
BY4743 diploid	YPD	0.11 to 1.1 nM, added at day 0	CLS: significant increase at 1 ng/mL	[80]
BY4742	SC	0.1 to 40 nM, added at day 0	CLS: significant increase at 20 and 40 nM	[65]
DBY2006	SD	200 nM, added at day 0	CLS: significant increase	[85]
BY4741	YPD and SD	1 pM to 10 μM, added at day 0	CLS: significant increase from 100 nM to 10 μM	[86]
BY4741 and S288C	YPD + hydroxyurea	10 nM, added at day 0	CLS: no difference	[87]
YPS128 and DBVPG6044	YPD	0.027 μM, added at day 0	CLS: significant increase	[27]
BY4741	SC	100 nM, added at day 0	CLS: significant increase	[88]

SD, synthetic defined; SC, synthetic complete; YPD, yeast peptone dextrose.

**Table 3:** The effect of resveratrol on yeast lifespan.

Strain	Medium	Dose/Treatment Time	Lifespan Effect	References
PSY316AT	YPD	10 to 100 µM, added at day 0	RLS: 70% increase at 10 µM CLS: no increase	[99]
K6001	SC	10 to 100 µM, added at day 0	RLS: significant increase at 10 µM	[100]
W303R, BY4742, PSY316	YPD	10 to 100 µM, added at day 0	RLS: no significant increase	[101]
PSY316AT	YPD	10 µM, added at day 0	RLS: 68% increase for derivative 5	[102]
EMY74.7	SC	10 to 200 mM, added at day 0	CLS: no significant increase	[103]
W303-1A	SC	100 µM, added at day 0	CLS: significant decrease	[104]
BY4741	SC	400 µM, added at day 0	Survival significant increase at 14 days	[105]
BY4742	SC	10 to 100 μM, added at day 0	CLS: decrease at 100 μM	[106]
BY4742 and *YAP1*	YPD	0.1 to 1000 μM, added at day 0	CLS: decrease at 100 and 1000 μM	[107]
BY4742	SD	0 to 200 μM	CLS Increased by 18%	[105]

**Table 4:** The effect of natural compounds on yeast lifespan.

Compound	Strain	Medium	Dose/Treatment Time	Lifespan Effect	Reference
Albanin A	BY4742	SD	7.5 to 120 μM	CLS: significant increase	[127]
Artocarpin	BY4742	SD	7.5 to 30 μM	CLS: significant increase	[127]
Astaxanthin	BY4741	YPD	10 to 50 μM, added at day 0	CLS: deletion strains increase 16–19% compared to wild-type	[128]
Astaxanthin	BY4742	YPD	250, 500, and 1000 µg/mL	CLS: significant increase	[129]
Beauveriolide I	BY4741	YPAD	40 μM, added at day 0	CLS: significant increase	[130]
Betulinic acid	BY4741	SD	0 to 100 μM, added after exponential phase	CLS: significant increase	[131]
Butein	*S. cerevisiae*	YPD	10 μM, added at day 0	CLS: significant increase	[99]
Caffeine	BY4741	SD	0.4, 0.8, 1 mM, exponential growth phase	CLS: significant increase	[132]
Calophyllum inophyllum	BY611	SD	1 mg/mL, added at day 0	CLS: significant increase	[133,134]
Citrus flavonoids	BY4742	SD	0.1, 1, 10, and 100 μM, initial inoculation (0 h)	CLS: significant increase	[135]
Cocoa polyphenol	*S. cerevisiae*	SD	0, 5 and 20 mg/mL, added at day 2	CLS: significant increase	[121]
Cocoa bean shell extract	*S. cerevisiae*	SD	0.05%, 0.1%, 0.2%	CLS: significant increase	[136]
Cryptotanshinone	BY4742	SD	20 nM to 5 μM, added at 0, 12, 24 h	CLS: 2.5 times increase	[137]
Cucurbitacin B	K6001 and BY4741	SD	0, 0.1, 0.3, or 1 μM	CLS: significant increase	[138]
Cudraflavone B	BY4742	SD	7.5 to 120 μM	CLS: significant increase	[127]
Cudraflavone C	BY4742	SD	3.75 to 60 μM	CLS: significant increase	[127]
Curcumin	BY4741	YPD	200 and 300 μM, added at day 0	CLS: significant increase	[139]
Ehretiquinone	K6001 and BY4741	YPD, SD	0, 1, and 3 µM, added at day 0	CLS: significant increase	[87,140]
Fisetin	*S. cerevisiae*	YPD	10 to 500 μM, added at day 0	CLS: 55% increase	[141]
Flavonoids	*S. cerevisiae*	YPD	10 µM, added at day 0	CLS: significant increase	[142]
Gentiopicroside	K6001 and BY4741	SD	0, 0.3, 1, 3, and 10 μM	CLS: significant increase	[143]
Ginsenoside Rg1	BY4742	YPD	100–250 µg/mL	CLS: significant increase	[144]
Glucosylglycerol	DBY746	SC	48 mM, 120 mM,	CLS: significant increase	[145]
Inokosterone	YOM36	SD	0, 1, and 3 μM, added at day 0	CLS: significant increase	[146]
Iron (FeSO4)	*S. cerevisiae*	SD	0, 50, 100, 200 μM,	CLS: significant increase	[147]
Leucine	EMY73	SC	30 µg/mL	CLS: significant increase	[148]
Lithocholic acid	BY4742	YPD	5 to 100 μM, added at day 0	CLS: significant increase	[149]
Magnolol	BY4741	YPD	1 mM, at the third day	CLS: significant increase	[128]
Metformin	*S. pombe*	SD	25 mM, added at day 0, and day 3	CLS: significant increase	[150]
Methionine sulfoxide	BY4743	SC	30 to 400 μM, added at day 0	CLS: 3.57% increase at 100 µM, 4.53% at 400 µM	[80]
Morusin	BY4742	SD	15 to 240 μM	CLS: significant increase	[127]
Mulberrin	BY4742	SD	60 μM	CLS: significant increase	[127]
Phosphatidylethanolamine	BY4741	SC	50 mM	CLS: significant increase	[151]
Polyalthia longifolia	BY611	SD	1 mg/mL, added at day 0	CLS: significant increase	[133]
Proline	*R. toruloides*	YEPD	2 mM, 5 mM, added at day 0	CLS: significant increase	[152]
Quercetin	W303-1A	DYNB	5 mM	CLS: significant increase	[57]
Salix alba extract (PE21)	BY4742	YEP	0.1%	CLS: significant increase	[153]
Spermidine	BY4741	SC	4 mM, stationary cultures (day 1)	CLS: significant increase	[154]
Tanshinones IIA, I	BY4742	SD	5 µM, 1.25 µM, added at 0, 12, 24 h	CLS: significant increase	[137]
Tschimganine	*S. pombe*	YPD	8.2 μM	CLS: significant increase	[155]

SD, synthetic defined; SC, synthetic complete; YPD, yeast peptone dextrose; YPAD, yeast extract peptone adenine dextrose; YEPD, yeast extract peptone dextrose; DYNB, Difco yeast nitrogen base.

**Table 5:** Genes involved in the extension of yeast CLS.

Gene	Strain	Medium	Mechanism of Action	Conservation in Other Species	Reference
ADH1	K2307; BY4741	YPD	Overexpression. reduces the acetaldehyde level, promotes the ethanol production.	*C. elegans*, *D. melanogaster*, mouse	[179,180,181,182,183]
ATG20	*S. pombe*; ED666, ED668	YES	Deletion of ATG20, functions in organelle autophagy.	-	[184,185]
CAR2	ED666, ED668; *S. pombe*	YES; SD	Deletion. Encodes ornithine transaminase and acts on amino acid metabolism.	*D. melanogaster*, mouse, porcine	[185,186,187,188,189]
ERG28	BY4741, BY4742; *S. pombe*, *S. cerevisiae*	YPAD; EMM, YE	Overexpression, involved in sterol synthesis.	Mouse, *Nothobranchius* fish, prostate cancer patients	[190,191,192,193,194]
GAS1	JY333, No.36	YE, SD	Mutation. Encodes cell wall 1,3-β-glucanosyltransferase, point mutation gas1-287 confer a long CLS.	*C. elegans*, mouse, human muscle stem cells	[195,196,197,198]
HSP104	ED666, ED668; *S. pombe*	YES	Deletion of HSP104 might induce Hsf1 activation.	*C. elegans*, mouse	[185,199,200,201]
NOP14	*S. cerevisiae*	YPD, YP-Gal	Overexpression. Ribosome regulation.	Mouse, human	[202,203,204]
KGD1	ED666, ED668	YES	Deletion causes CLS extension.	-	[185]
KSP1	ED666, ED668; *S. cerevisiae*	YES; YPD, YP-Gal, SMD, SD-N	Deletion. Regulated by PKA and activates TORC1.	-	[185,205]
LCF1 and LCF2	JY333, TK107; JY333, JY336, JY741	SD; YPD	Overexpression of LCF1, deletion of LCF2.	-	[206,207]
LYS7	ED666, ED668	YES	Deletion. Involved in lysine biosynthesis.	*C. elegans*	[185,208]
MOC3	ED666, ED668; *S. pombe*	YES	Deletion, physically interacts with Kgd1.	-	[185,209]
NBR1	*S. pombe*	YES	Deletion.	Mouse	[184,210]
NNK1	JY333	SD, YE	Nonsense mutation of NNK1 (nnk1-35), associated with TOR.	-	[211]
OGA1	*S. pombe*, *S. cerevisiae*; *S. pombe*	EMM, YE; SD, EMM	Overexpression. Involved in the TOR pathway and ribosome control.	*C. elegans*	[191,212,213]
PDC201 and PDC202	972, ED665, JH43, ESX5, JY01-JY11	EMM, YE, ME	Overexpression. The transcription factor Phx1 may induce Pdc201 and Pdc202, both of which encode pyruvate decarboxylase.	-	[214]
PHT1	Y8205, YEG01-CFP, YEG01-RFP, BY4741	SMD, YNB-If, SD-N, YPD, YPEG	Deletion, parallel with the Pmk1 and Sty1 pathways.	Mouse, porcine, cancer patients	[215,216,217,218]
PMA1	JY333; S288c (BY)	SD; YEPD	Mutation (pma1-L16, pma1-L18), loss of function mutations (D138N and A270D) of Pma1, functional decline in Pma1.	-	[219,220]
SDH1	ED666, ED668; *S. pombe*, *S. cerevisiae*	YES; EMM, YE	Overexpression, encodes succinate dehydrogenase.	-	[185,191]
SPCC18.02	*S. pombe*, *S. cerevisiae*;	EMM, YE	Overexpression of SPCC18.02, encode a transmembrane transporter protein, parallel with the Pmk1 pathway.	-	[191]
CLG1	*S. pombe*	YES. EM, SD	Deletion of CLG1, encodes a cyclin like protein.	-	[221]
ECL1, ECL2, and ECL3	*S. pombe*	-	Overexpression.	-	[222]
PMK1, PEK1, and MKH1	JY333, No.36	YE, SD	Deletion.	-	[195]
PKA and STY1	*S. pombe*	-	Deletion. Inhibition of the PKA pathway, whereas activation of the Sty1 pathway.	-	[180,223]
GIT5	*S. pombe*	-	Both overexpression and deletion. Suppresses the glucose-signaling pathway.	-	[224]
GRX4	*S. pombe*	-	Overexpression.	-	[224]
PHP2, PHP3, and PHP5	JY333, HM3802, PR110, RM1, RM3, KS1376, TK107	YE, EMM, SD	Deletion. The suppression of the Php complex activates the Sty1 pathway.	-	[225]
PAR1	ED666, ED668; haploid YKO collection	YES; YPD	Deletion. Encodes a protein phosphatase 2A B’-regulatory subunit and may be involved in the TORC1 pathway.	-	[185,226]
PDB1	*S. pombe*, *S. cerevisiae*;	EMM, YE	Overexpression. Encodes a subunit of pyruvate dehydrogenase.	-	[191]
PPI1	*S. pombe*, *S. cerevisiae*;	EMM, YE	Overexpression. Encodes cyclophilin.	-	[191]
*REB1*	PJ69-4A	LB	Deletion. Encodes RNA polymerase I transcription termination factor.	-	[227]
*RPB10*	BY4741	YPD	Overexpression.	-	[180]
*SCK1*	*S. Cerevisiae*, *S. pombe*	YES	Overexpression. Positively interacts with Sty1 and Tim18.	-	[228]
*SHD1*	ED666, ED668	YES	Deletion.	-	[185]
*TPS0*	*S. pombe*, *S. cerevisiae;*	EMM, YE	Overexpression. Encodes mitochondrial lipid translocator protein and negatively interact with Tor1.	-	[191]
*UCK2*	*S. Cerevisiae*, *S. pombe*	YES	Deletion. Encodes uracil phosphoribosyltrans-ferase and negatively interact with Reb1.	-	[228]
*UFD2*	ED665	YE, EMM	Deletion. Encodes ubiquitin-protein ligase E4. Positively interacta with Git3 and Git5, and may be involved in the PKA pathway.	*C. elegans*	[229,230]
*URE4*	ED666, ED668	YES	Deletion.	-	[185]
*VMA1*	*S. pombe*	SD, YES	Overexpression. Encodes the subunit A of vacuolar ATPase and regulates vacuolar acidification.	-	[231]
*SPAC323.03c*	ED666, ED668	YES	Deletion. Negatively interacts with Par1.	-	[185]
*SPBP4H10.16c*	ED666, ED668	YES	Deletion.	-	[231]
*SPCC18.02*	*S. pombe*, *S. cerevisiae;*	EMM, YE	Overexpression. Negatively interacts with Mkh1.	-	[191]

SD, synthetic defined; SC, synthetic complete; YPD, yeast peptone dextrose; YES, yeast extract plus supplements; YPAD, yeast extract peptone adenine dextrose; EMM, Edinburgh minimal medium; YE, yeast extract; YP-Gal, galactose-containing medium; SMD, synthetic medium; SD-N, starvation medium; ME, malt extract medium; YEPD, yeast extract peptone dextrose; LB, Luria–Bertani medium.

## Data Availability

No new data were created or analyzed in this study.

## Abbreviations

The following abbreviations are used in this manuscript:

RLS	Replicative lifespan
CLS	Chronological lifespan
ER	endoplasmic reticulum
CFUs	colony-forming units
PI	Propidium iodide
PI-FCM	Propidium iodide (PI) staining combined with flow cytometry
OD	Optical density
SGA	Synthetic genetic array
IPT	intercellular protein transfer
SASP	senescence-associated secretory phenotype
Sis	Survival integrals
CR	Calorie restriction
DR	dietary restriction
QTL	Quantitative trait loci
BCAAs	Branched side chain amino acids
GAAC	General amino acid control
NEAAs	Nonessential amino acids
NCR	Nitrogen catabolite repression
GABA	c-aminobutyric acid
FKBP12	FK-binding protein 12
TOR	Target of rapamycin
ITP	Interventions Testing Program
STACs	Sirtuin-activating compounds
NAD^+^	Nicotinamide adenine dinucleotide (oxidized form)
5-DN	5-demethylnobiletin
5-DT	5-demethyltangeretin
ROS	Reactive oxygen species
Atg	Autophagy-related genes
SOD	Superoxide dismutase
SAGA	Spt-Ada-Gcn5 acetyltransferase
GTP/GDP	Triphosphate/guanosine diphosphate
AC	Adenylate cyclase
PKA	Protein kinase A
SIR	Silent information regulator
